# Isolation of mineralizing Nestin+ Nkx6.1+ vascular muscular cells from the adult human spinal cord

**DOI:** 10.1186/1471-2202-12-99

**Published:** 2011-10-10

**Authors:** Daria Mamaeva, Chantal Ripoll, Claire Bony, Marisa Teigell, Florence E Perrin, Bernard Rothhut, Ivan Bieche, Rosette Lidereau, Alain Privat, Valérie Rigau, Hélène Guillon, Florence Vachiery-Lahaye, Daniele Noel, Luc Bauchet, Jean-Philippe Hugnot

**Affiliations:** 1 CNRS UPR 1142, Institute of Human Genetics, 141, rue de la Cardonille, 34396 Montpellier Cedex 05, France; 2 INSERM U1051, Institut des Neurosciences de Montpellier, Hôpital St ELOI, BP 74103 80, av Augustin Fliche, 34091 Montpellier Cedex 05, France; 3 INSERM U844, Institut des Neurosciences de Montpellier, Hôpital St ELOI, BP 74103 80, av Augustin Fliche, 34091 Montpellier Cedex 05, France; 4 IKERBASQUE, Basque Foundation for Science, 48011 Bilbao, Spain; 5 Department of Neuroscience, Faculty of Medicine and Odontology, University of the Basque Country UPV/EHU, 48940 Leioa, Spain; 6 INSERM U735, Institut Curie - Hôpital René Huguenin, 35 rue Dailly, 92210 Saint-Cloud, France; 7 CHU Montpellier, Hopital Guy de Chaulliac, Montpellier, France; 8 Université Montpellier 2, Place Eugène Bataillon, 34095 Montpellier cedex 5, France

**Keywords:** human central nervous system, spinal cord, stem cells, vascular muscle cells, osteogenesis, Nkx6.1, calcification

## Abstract

**Background:**

The adult central nervous system (CNS) contains different populations of immature cells that could possibly be used to repair brain and spinal cord lesions. The diversity and the properties of these cells in the human adult CNS remain to be fully explored. We previously isolated Nestin^+ ^Sox2^+ ^neural multipotential cells from the adult human spinal cord using the neurosphere method (i.e. non adherent conditions and defined medium).

**Results:**

Here we report the isolation and long term propagation of another population of Nestin^+ ^cells from this tissue using adherent culture conditions and serum. QPCR and immunofluorescence indicated that these cells had mesenchymal features as evidenced by the expression of Snai2 and Twist1 and lack of expression of neural markers such as Sox2, Olig2 or GFAP. Indeed, these cells expressed markers typical of smooth muscle vascular cells such as Calponin, Caldesmone and Acta2 (Smooth muscle actin). These cells could not differentiate into chondrocytes, adipocytes, neuronal and glial cells, however they readily mineralized when placed in osteogenic conditions. Further characterization allowed us to identify the Nkx6.1 transcription factor as a marker for these cells. Nkx6.1 was expressed in vivo by CNS vascular muscular cells located in the parenchyma and the meninges.

**Conclusion:**

Smooth muscle cells expressing Nestin and Nkx6.1 is the main cell population derived from culturing human spinal cord cells in adherent conditions with serum. Mineralization of these cells in vitro could represent a valuable model for studying calcifications of CNS vessels which are observed in pathological situations or as part of the normal aging. In addition, long term propagation of these cells will allow the study of their interaction with other CNS cells and their implication in scar formation during spinal cord injury.

## Background

It is now well established that the CNS contains a pool of neural stem and progenitor cells that are either located in specific locations called niches or dispersed in the parenchyma, notably in the white matter. These cells can be isolated using a cell sorting strategy based on the expression of markers such as CD133 and A2B5. They typically grow on non adherent substrates when they generate clonal expansions called neurospheres [1], which on differentiation, generate glial cells and neurons. This endogenous pool of immature cells is activated and recruited in several CNS pathologies such as stroke or degenerative diseases in an attempt to compensate for the cellular loss. Much of our knowledge on these cells is derived from studies performed in rodents, but much less is known on these stem cells in human. Adult neural stem and progenitor cells have been isolated from different parts of the human brain, namely the olfactory bulb, the subventricular zone, the hippocampus, the cortex, using the neurosphere assay [2-9]. More recently, using adherent conditions and a specific media, adult human neural precursors have been isolated from the human temporal cortex and the hippocampus [10]. These cells express glial markers and are able to generate both glial and mature neuronal cell types in vivo and in vitro. They could represent a potential endogenous cellular source that could be used to alleviate neuronal loss which occurs in many CNS diseases. However, much more work is needed to isolate and characterize these cells from different human CNS locations and, in particular, to analyse the signalling mechanisms leading to their differentiation.

Besides neural stem cells, the adult CNS might also contain a pool of mesenchymal stem cells (MSC) which appear to differentiate into mesenchymal lineage cells (adipocytes, osteoblasts and chondroblasts) and possibly neural lineage cells. However this has mainly been observed in mice [11] and the actual differentiation capacity of these cells remains to be validated in detail.

Compared to brain, little is known about neural precursors in the adult spinal cord [12]. We recently isolated multipotential cells in the human adult spinal cord using the neurosphere method [13]. In this article, we set out to explore the possibility of isolating adult neural stem/progenitor cells from the adult human spinal cord using adherent conditions previously described [10]. We found that these conditions mainly result in the propagation of mesenchymal cells expressing Snai2 and Twist1 and with the hallmarks of smooth muscle muscular cells. These cells do not appear to be MSC, nor neural stem cells, but they can readily mineralize in vitro when placed in appropriate conditions. Additional characterization of these cells allowed us to identify the Nkx6.1 homeodomain transcription factor as a marker for these cells.

## Results

### Isolation of Nkx6.1^+ ^vascular muscular cells from adult human spinal cord

**Figure 1:**
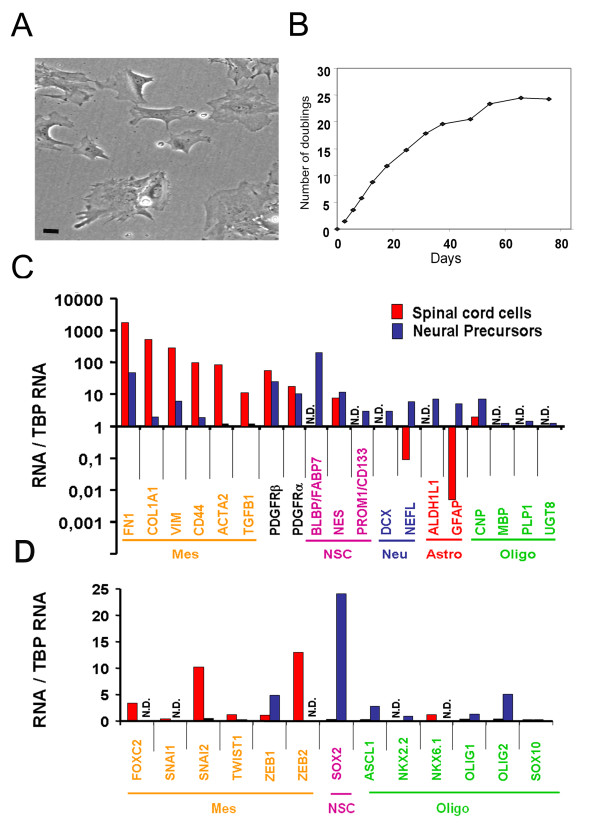
**Characterization of proliferating cells isolated from human adult spinal cord**. **A: **Phase contrast photograph of cells (passage 4) isolated from the human spinal cord. Most cells display a mesenchymal morphology. Scale bar = 10 μm. **B**: Proliferation rate of adherent human cells. Y axis represents the cumulative number of doublings measured at each passage. **C, D: **QPCR analysis of gene expression in spinal cord cells (red bars) and human fetal brain neural stem cells cultured for 3 passages in serum-containing spinal cord cell medium (blue bars). Y axis represents the expression ratio between the indicated messenger and TBP (TATA binding protein) mRNA. **C**: Mesenchymal and neural markers. **D**: Mesenchymal and neural transcription factors. Mes: mesenchymal. NSC: neural stem cells. Neu: neurons. Astro: astrocytes. Oligo: oligodendrocytes. N.D: Not detected.

**Figure 2:**
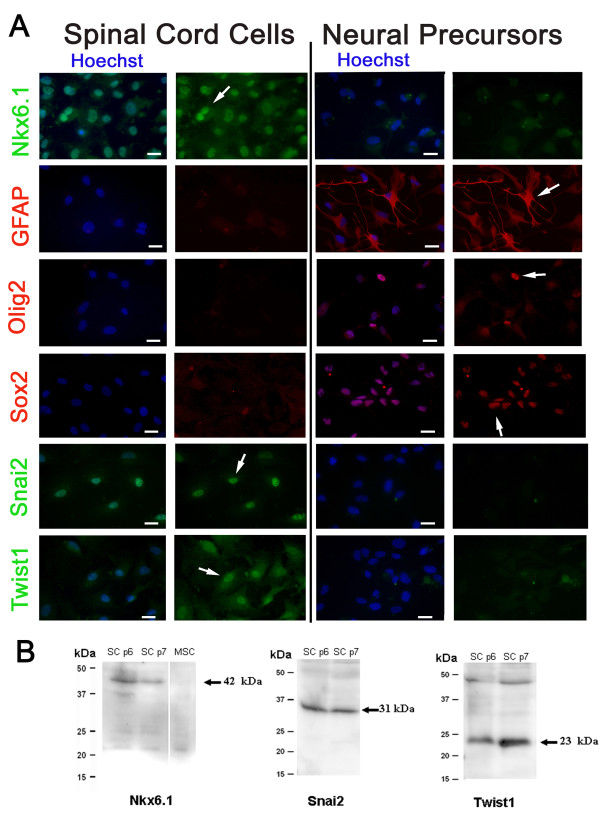
**Immunofluorescence characterization**. **A: **Spinal cord cells and human fetal neural stem cells (cultured for 3 passages with serum-containing spinal cord cell medium) were labelled with indicated antibody. Arrows show examples of stained cells. Scale bars = 10 μm. **B: **Western blot analysis for indicated antibodies. A main band at the expected size is observed for the three antibodies in spinal cord stem cells. SC p6, SC p7: spinal cord cells analysed at passages 6 and 7. MSC: human mesenchymal stem cells isolated from bone marrow.

To isolate human neural stem cells using adherent condition as used in [10], human spinal cord was rapidly processed as previously described [13] and seeded with neural stem cell medium containing serum and EGF/FGF. Under these conditions, cells with a mesenchymal morphology (Figure 1A) grew rapidly and could be propagated for approximately 3 months (approximately 25 divisions and 9-10 passages) before undergoing proliferation decrease, probably as a consequence of cellular senescence (Figure 1B). The cells were characterized at passage 4 by QPCR and immunofluorescence. We first measured the expression of genes typically expressed by cells of the neural lineage, i.e astrocytes (*Aldh1l1, Gfap*), oligodendrocytes (*Cnp, Mbp, Plp1, Sox10, Ugt8*), oligodendrocyte progenitor cells (*Ascl1, Nkx2.2, Nkx6.1, Olig1/2, Pdgfrα/β*), neurons (*Dcx, Nefl*) and stem cells (*Fabp7/Blbp, Nestin, Prominin/CD133, Sox2*). Surprisingly, although these cells were derived from the CNS, we found that the expression of these genes was either low or undetectable (Figure 1C, D) and only *Nestin, Cnp *and *PdgfRα/β *could be detected with less than 25 cycles of QPCR. We confirmed by immunofluorescence the absence of detectable expression of Sox2, GFAP and Olig2 which are well documented markers for neural stem cells and glial cells (Figure 2A). We then questioned the presence of markers frequently expressed in mesenchymal cells (*Acta2, CD44, Col1A1, Fn1, Vim, Tgfβ1*), bearing in mind that these can also be found expressed in cells of the neural lineage, and mesenchymal transcription factors (*FoxC2, Snai1/2, Twist1, Zeb1/2*). In sharp contrast with the neural lineage genes, the expression of these genes was readily detected (Figure 1C, D). We confirmed by immunofluorescence and western blot that Snai2 and Twist1 were expressed at the protein level (Figure 2A, B). These two transcription factors were detected in the nuclei of most cells (>80%) (Figure 2A).

The possibility existed that the isolated cells were derived from the in vitro differentiation of endogenous spinal neural precursor cells which would adopt a mesenchymal phenotype when cultured in serum-containing medium. To explore this hypothesis, we cultured human fetal brain neural stem cells in this medium for three passages and then explored the phenotype of these cells by QPCR and immunofluorescence (Figure 1C, D, 2A). In contrast with cells isolated from the adult spinal cord, our immunofluorescence analysis revealed that these cells expressed a high level of several typical neural markers such as GFAP, Sox2, Olig2 whereas Snai2 and Twist1 were not detectable (Figure 2A). QPCR confirmed these results and further indicated the maintenance of several typical markers for neural cells (for instance *CD133/Prom1, Dcx, PLP1 *respectively for stem, neuronal and oligodendrocytic cells) even when cultured in serum-containing media (Figure 1C, D).

**Figure 3:**
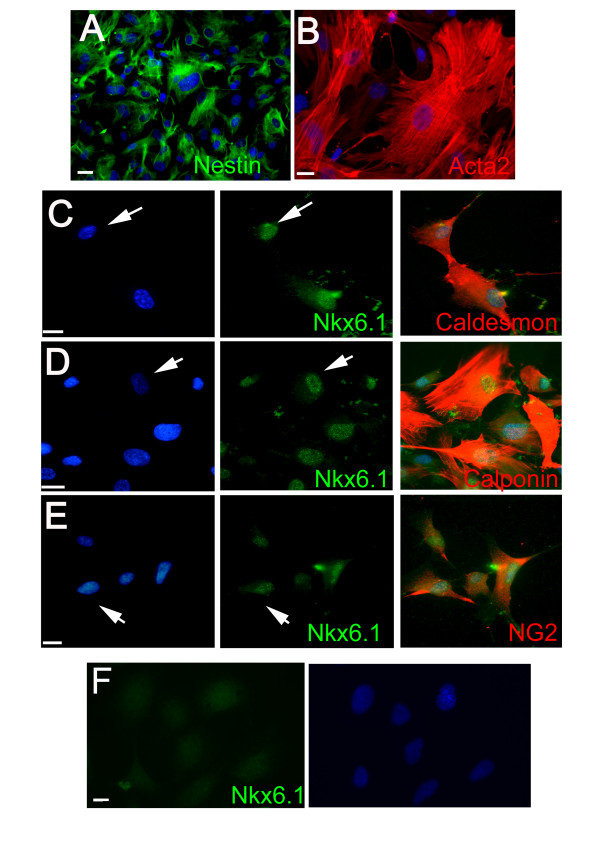
**Expression of smooth muscle markers and Nkx6**.1 in spinal cord cells. **A-F: **Immunofluorescence staining of spinal cord cells (**A-E**) and human mesenchymal stem cells (**F**). The vast majority of cells (>80%) express Nestin, Acta2, Caldesmon, Calponin and NG2. Nuclear Nkx6.1 staining is detected in Caldesmon^+^, Calponin^+^, NG2^+ ^spinal cord cells (**C, D, E**) but not in human mesenchymal stem cells (**F**). Scale bars = 10 μm.

The high expression of transcripts for smooth muscle actin (*Acta2*) in spinal cord cells suggested that the culture contained a majority of vascular muscular cells, an assumption consistent with the detection of *Nestin *and *PdgfRα/*β (Figure 1C), two markers also expressed by vascular cells [14,15]. In order to confirm this possibility, we explored by immunofluorescence the expression of Acta2 and Nestin, together with Calponin, Caldesmon and NG2, three other markers of vascular muscular cells. The vast majority of the cells (>80%) were positive for these five proteins, confirming that these cultures mostly contained vascular muscular cells (Figure 3A-E).

**Figure 4:**
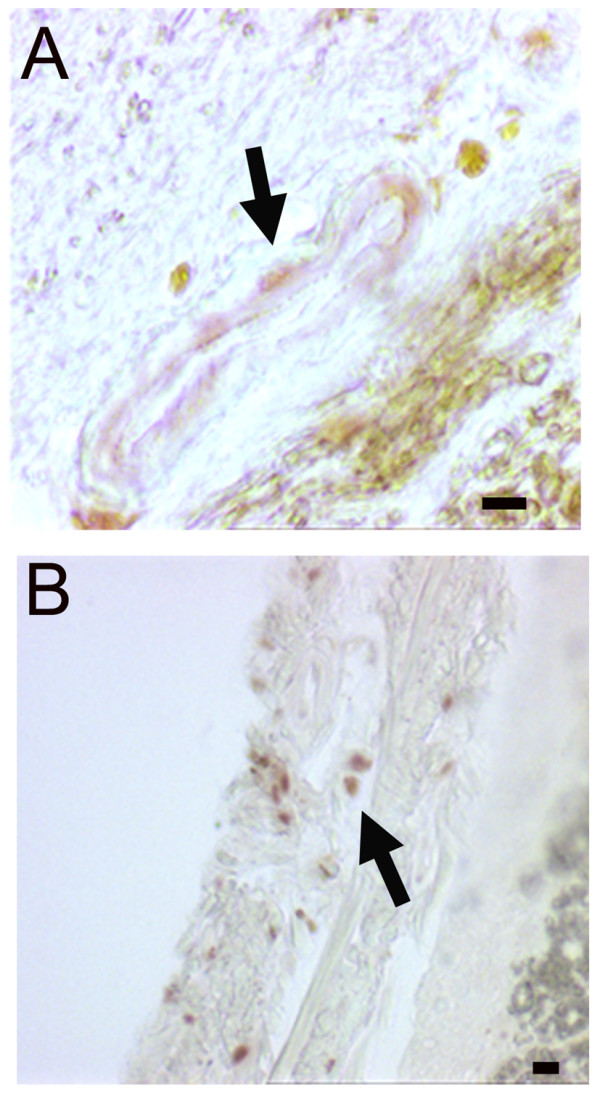
**Nkx6.1 expression in human spinal cord**. **A**: Example of detection of nuclear Nkx6.1 staining (arrow) in a vessel in the human spinal cord parenchyma. **B**: Example of detection of nuclear Nkx6.1 staining in a vessel (arrow) in the meninges. Scale bars = 10 μm.

One unexpected transcription factor detected in the culture by QPCR was Nkx6.1 (Figure 1D). This homeodomain protein is implicated in the ontogenesis of oligodendrocytes and of pancreatic cells [16,17] but its expression in vascular cells has not been reported in adults. The presence of Nkx6.1 protein was confirmed by immunofluorescence and western blot in these cultures (Figure 2A, B). The vast majority of the cells (>80%) showed a clear nuclear staining and these cells coexpress Calponin, Caldmesmon and NG2 (Figure 3C, D, E). In contrast, Nkx6.1 was not detected in human fetal neural stem cells cultured in the same conditions for three passages (Figure 2A). The expression of Nkx6.1 by vascular cells was then confirmed in vivo on adult human spinal cord sections. Nkx6.1^+ ^cells were detected around large vessels in the meninges and in the parenchyma (Figure 4A, B). As previously reported [13], Nkx6.1 was also expressed by ependymal cell of the central canal (data not shown). We concluded from these data that adherent cultures derived from adult human spinal cord led to the isolation of a cellular population mainly composed of vascular muscular cells that express the homeodomain protein Nkx6.1 and the mesenchymal transcription factors Snai2 and Twist1.

### Nkx6.1^+ ^cells are not multipotential stem cells but mineralize *in vitro*

**Figure 5:**
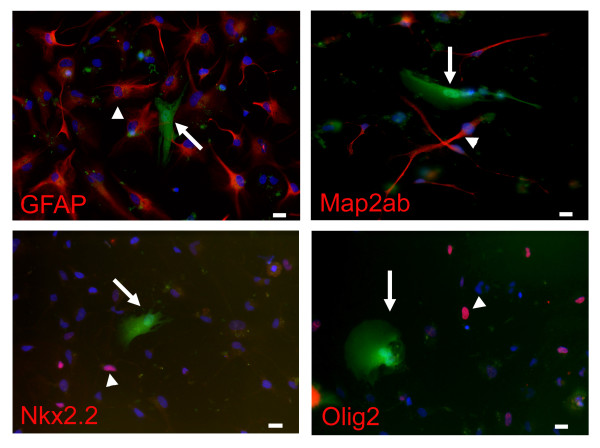
**Co-culture of spinal cord cells with differentiated human fetal brain neural stem cells**. Spinal cord cells labelled with GFP-expressing lentivirus (green fluorescence, arrows) do not stain with GFAP, Map2ab, Olig2 and Nkx2.2 antibodies (red) in contrast to neural precursor cells (arrowheads). Scale bars = 10 μm.

It has recently been reported that adult stem cells with mesenchymal features located in various tissues could behave as multipotential stem cells [11,18-21]. These cells could give rise to cells of several lineages including neurons and myelinating cells. We thus explored the multipotentiality of Nkx6.1^+ ^cells by placing them in different media and culture conditions. First, by culturing them in non adherent conditions in defined neural stem cell medium, these cells were not able to form neurospheres, a hallmark of neural stem cells. Second, we further examined the possibility of a neural differentiation by labelling the cells with a GFP-expressing lentivirus, then by coculturing them for 2 weeks with human young neurons, obtained by differentiation of human fetal brain neural stem cells. In these conditions, we could not detect staining of GFP-labelled cells (100 individual cells examined) with neuronal (Map2ab), astrocytic (GFAP) or oligodendrocytic (Olig2, Nkx2.2) markers, strongly indicating the absence of neural differentiation of these cells (Figure 5). Third, we treated the cells with BMP2, BMP4, BMP7, CNTF, CNTF/BMP4, LIF, LIF/BMP4 or T3 to promote an astrocytic [22,23] or an oligodendrocytic fate [24] respectively, but again no differentiation was observed (data not shown).

**Figure 6:**
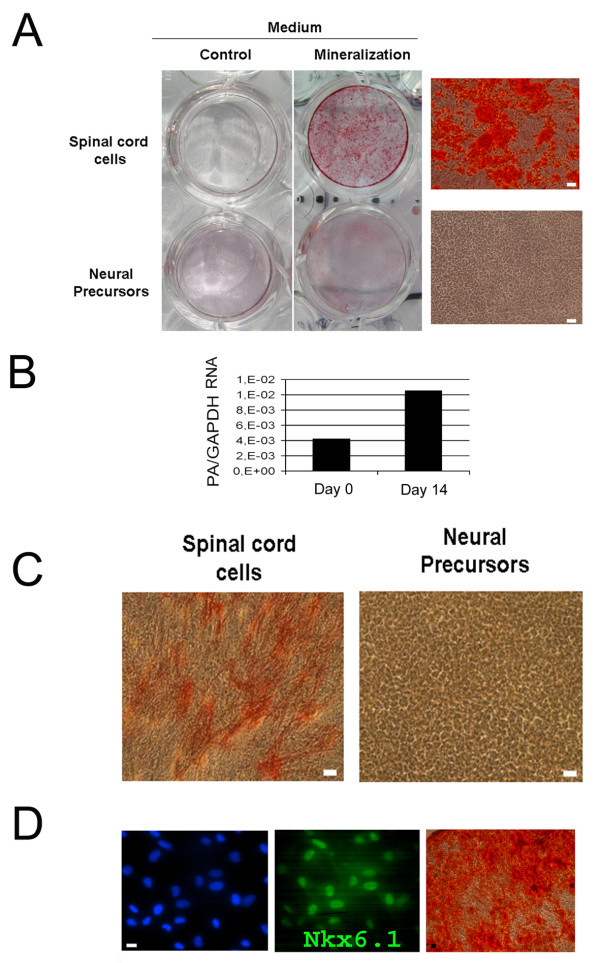
**Mineralization of spinal cord cells**. **A: **Alizarin red S stainings performed on spinal cord cells and on human fetal brain neural stem cells (cultured for 3 passages in serum-containing spinal cord cell medium). Cells were incubated for 14 days in control (left-hand wells) or in osteogenic medium (right-hand wells). High-magnification photographs of corresponding wells are presented on the right-hand side. Scale bars = 10 μm. **B: **QPCR detection of alkaline phosphatase mRNA in spinal cord cells after 0 and 14 days of differentiation in osteogenic media. Y axis represents the ratio between alkaline phosphatase mRNA (PA) and GAPDH mRNA. This experiment is representative of three independent experiments. **C**: Detection of alkaline phosphatase activity with naphtol ASBI phosphate/Na3-fast red staining in spinal cord cells and human fetal neural stem cells placed in osteogenic media for 14 days. No staining is detected in neural precursors. **D**: Characterisation of one clone, representative of 34, derived from the primary human spinal cord culture. Nkx6.1 is detected in the nuclei of most cells. Cells placed in osteogenic differentiation medium for 21 days stained with Alizarine red S. Scale bars = 10 μm.

Considering the mesenchymal features of these cells and as vascular muscular cells have been reported to be endowed with stem cell properties [18,20] we next examined the capacity of Nkx6.1^+ ^cells to generate chondrocytes, adipocytes and osteoblasts, three lineages commonly produced by mesenchymal stem cells. The cells were placed in adequate media, and the differentiation was monitored by staining with Oil red O (adipogenesis), Alizarine red S (osteogenesis) and Alcian blue (chondrogenesis) and by QPCR for markers for adipocytes (Lpl, Fabp4, PPARg), chondrocytes (Col2b, Col10, Aggregan, Mmp13, Comp, Sox9) and osteoblasts (SPARC, Osteocalcin, Runx2, phosphatase alkaline). As a control, the differentiation of human mesenchymal stem cells isolated from bone marrow was monitored in parallel. Under these conditions, no differentiation towards chondrocytes or adipocytes was observed (not shown). Only deposition of calcium phosphate precipitates, as observed with Alizarine red S staining (Figure 6A), could convincingly be detected. This was accompanied by a sharp increase of alkaline phosphatase mRNA (Figure 6B) and activity which was detected with Naphthol ASBI phosphate and Fast Red Trisodium staining (Figure 6C). No increase of the other osteoblast markers compared to that obtained with bone marrow mesenchymal stem cells could be demonstrated (not shown) suggesting absence of complete differentiation towards osteoblasts. Equally, we could not detect the expression of Nkx6.1 by western blot (Figure 2B) and immunofluorescence (Figure 3F) in bone marrow mesenchymal stem cells, suggesting that CNS Nkx6.1^+ ^vascular cells are weakly related to these cells. We also examined the capacity of human fetal neural stem cells cultured in the presence of serum to mineralize in the same conditions. Figure 6A, C shows however that these cells were unable to do so.

Finally, to ascertain that mineralization was produced by Nkx6.1^+ ^cells and not by other contaminating cell types, we performed single cell cloning in 96 wells to isolate Nkx6.1^+ ^cell clones which were then placed in mineralizating conditions. Among 34 isolated Nkx6.1^+ ^clones, 95% of them were able to mineralize (Figure 6D).

We concluded from these studies that Nkx6.1^+ ^vascular muscular cells isolated from the adult CNS do not behave as neural or mesenchymal stem cells but can readily mineralize *in vitro*.

## Discussion

Neural stem cells are generally isolated and cultured using non adherent conditions and defined medium to generate free-floating aggregates, the so-called neurospheres. Adherent cultures with serum-containing medium have also been successfully used to isolate and culture adult neural stem cells from adult human brain (temporal lobe). We previously reported the isolation of human spinal cord stem/progenitor Sox2^+ ^cells using the neurosphere method [13], however we were unable to identify such cells using adherent conditions in the present study. Considering the low number of these cells as compared to other spinal cord cells, it is possible that these cells are rapidly diluted during the culture passages. Alternatively it is conceivable that the presence of serum leads to their premature differentiation.

We report here that, in contrast to neural stem/progenitor cells, these conditions lead to the isolation of a cellular population mainly composed of Nkx6.1^+ ^vascular muscular cells. Although these cells express some markers for neural and mesenchymal stem cells, such as Nestin or CD44, they do not behave as multipotential stem cells, at least in the conditions we employed. In fact, only differentiation along the osteogenic lineage, as evidenced by their ability to mineralize *in vitro*, was observed. The calcification of brain vessel muscle cells has been observed since 1884 [25,26] in pathological situations or as part of the brain aging process [27]. In elderly brains, mild calcification of vessels and parenchyma is a normal and common incidental finding in the basal ganglia, pineal gland, choroid plexus, dura and habenula. This vasculopathy may account for the sclerosis of the aged hippocampus and the concomitant loss of CA1 neurons [28].

There is also now clear evidence for a close association between vasculature and neurogenesis [29], the so-called vascular stem cell niche, and the observation of an age-related neurogenesis decline [30] may be related to some modifications of vascular cell properties. In addition, recent work by Göritz C, et al [31] demonstrated a critical role of vascular cells in the formation of the scar following spinal cord injury. The extensive growth of Nkx6.1^+ ^human vascular muscular cells under our conditions will allow further studies on their interactions with adult neural stem cells, their role in the aging process and their implication in the scar formation.

Nkx6.1 is a homeodomain protein that plays a major role in CNS development, notably in spinal cord [32]. Being a target of the ventralizing SHH morphogen, Nkx6.1 is required for motoneuron generation and correct oligodendrogenesis. In agreement with the detection of Nkx6.1 in adult spinal cord meninges (Figure 4), Nkx6.1 is also expressed in ventral meninges during spinal cord development [33]. Outside the CNS, Nkx6.1 is required for pancreatic β-cell development and its disruption results in 90% decrease of these cells [16]. Nkx6.1 is also detected in esophageal and tracheal smooth muscle cells during development [33]. Consistent with this observation, we report here that in the adult human spinal cord, vascular smooth muscle cells express this transcription factor in vitro and in vivo. Its role in these cells remains to be explored. As Nkx6.1 is implicated in the positive regulation of β-cell proliferation [34], notably by direct interaction with the cyclin A2 and B1 promoters, a similar function could be hypothesized for CNS vascular muscular cells proliferation.

## Conclusions

In this study we have isolated vascular cells from the human adult spinal cord and showed that these cells are neither endowed with mesenchymal nor neural stem cell properties. We demonstrated that these cells express the developmental homedomain protein Nkx6.1, two mesenchymal transcription factors (Snai2 and Twist1) and that they can readily mineralize when placed in osteogenic conditions. These cells constitute a useful model for studying CNS vessel calcification in vitro.

## Methods

### Cell culture

Human spinal cord was obtained from one organ-donor patient (40 years old, accidental death) in strict agreement with the French bioethics laws (articles L1232-1 to L1232-6) and after approval by the French institution for organ transplantation. An informed consent from the family was obtained by the organ procurement organization (OPO) for this study. A sample of the lumbar spinal cord was removed and rapidly processed for cell culture as previously described [13]. Cells were cultured at approximately 4 × 10^3^ per cm^2 ^cell culture flasks in the presence of DMEM/F12 with L-glutamine complemented with N2 supplement (Fischer Scientific, http://www.fishersci.com), 35 μg/ml of bovine pituitary extract (Fischer Scientific), 5% of certified fetal calf serum (Fischer Scientific), 20 ng/ml of human EGF and FGF2 (Peprotech, http://www.peprotech.com), heparin (2 μg/ml), ciprofloxaxine (2 μg/ml), gentamycin (10 μg/ml) (Fischer Scientific), fungizone (0.25 μg/ml) (Fischer Scientific), fungin (10 μg/ml) (Invivogen, http://www.invivogen.com). Media was replaced twice a week and cells were passaged at 80-90% confluency using a 3 min dissociation step at 37°C with Trypsin/EDTA (Fischer Scientific) containing 0.25% Trypsin. Cells were frozen in complete media containing 10% DMSO. For cloning experiments (Figure 6D), the cell culture was dissociated and single cell seeding in 96 well plates was performed using an Aria cytometer (BD, http://www.bdbiosciences.com) equipped with an automated single cell deposition device. After 3 weeks, each clone was split in two and expanded to perform Nkx6.1 staining and osteogenic differentiation.

### Differentiation into neurons, astrocytes and myelinating cells

To assess the capacity of human adult spinal cord cells to differentiate into oligodendrocytic, astrocytic or neuronal cells, cells were seeded on differentiated human fetal brain neural stem cells. The latter were obtained by growing human fetal brain neural stem cells (16 weeks of gestation, Lonza, http://www.lonza.com) as neurospheres then by differentiating them on laminin-coated coverslips to generate young neurons and glial cells according to the manufacturer's protocols. Spinal cord human cells were pre-labelled using an infection with a GFP-expressing lentivirus to distinguish them from fetal brain cells. Cells were co-cultured for 1-2 weeks before assessing their differentiation into glial and neuronal cells using neural lineage markers (Map2ab, Gfap, Olig2, Nkx2.2). To assess the capacity of the spinal cord cells to differentiate into astrocytes, cells were treated by BMP2 (10 ng/ml), BMP4 (10 ng/ml), BMP7 (10 ng/ml), LIF (10 ng/ml), LIF + BMP4, CNTF (10 ng/ml) or CNTF+BMP4 for 4 days before staining for GFAP. For oligodendrocytic differentiation, cells were treated for one week with 15 nM Triiodo-L-Thyronine (T3) (Sigma, http://www.sigmaaldrich.com) + N2 supplement (Fischer Scientific, http://www.fishersci.com) or Neurobasal media with B27 and vitamin A, before staining for GalC and O4 antigens.

### Differentiation into adipocytes, chondroblasts and osteoblasts

As a control, the differentiation of human mesenchymal stem cells isolated from bone marrow as described in [35] was monitored in parallel with spinal cord cells and human fetal neural stem cells. For adipogenic differentiation, cells were plated at a density of 8 × 10^3 ^cells/cm^2 ^in DMEM-F12 containing 5% newborn calf serum, 1 μM dexamethasone, 50 μM isobutyl-methylxanthine and 60 μM indomethacin. Stimulation was carried out for 3 weeks with the medium changed every 2-3 days. Adipocyte differentiation was confirmed on day 21 by visualization of lipid droplets by oil red O staining and QPCR using primers as described in [35]. For chondrogenic differentiation, cells were cultured in 15 ml polypropylene conical tubes with 2.5 × 10^5 ^cells, after centrifuging for 5 min at 600 g. The resulting pellets were cultured in 500 μl DMEM supplemented with 0.1 μM dexamethasone, 0.17 mM ascorbate-2 phosphate, 1% insulin-transferrin-sodium selenite supplement and 10 ng/ml of recombinant TGFβ3 (R&D Systems). Media were changed every 2-3 days. Differentiation was assessed on samples on day 21 by QPCR using specific primers [35]. Osteogenesis was induced in spinal cord cells and human fetal brain neural stem cells by culturing at low density (2.5 x10^3 ^cells/cm^2^) in osteogenic medium consisting of DMEM (high glucose) with 10% fetal calf serum, 10 mM β-glycerophosphate, 0.1 μM dexamethasone, 0.05 mM ascorbic acid (Sigma) and NaH_2_PO_4 _3 mM. Medium was changed every three days for 2 weeks. Osteoblast differentiation was assessed on day 14 by visualization of matrix calcification by Alizarin red S staining and QPCR as previously described [35]. Phosphatase alkaline activity detection by naphthol ASBI phosphate and Fast Red Trisodium staining was performed as previously described [36]. In this condition, cells were cultured for 2 weeks in osteogenic medium but without NaH_2_PO_4_.

### Histology and immunodetection

For paraffin sections, human lumbar spinal cord samples were fixed in formaldehyde-acetic acid-methanol (10%-10%-40% v/v) for 72 h, embedded in paraffin, then processed for antigen demasking by boiling for 40 min in EDTA 10 mM pH 7.2. Immunoperoxydase detection (diaminobenzidine, Dako) was performed as previously described [13] on 4 μm paraffin sections. Immunodetection was performed on cell cultures as previously described in [37]. Nuclei were stained with 2 μg/ml Hoechst 33242. The following primary antibodies were used: Acta2 (1:100, mouse monoclonal, 1A4, Sigma), Caldesmon (1:250, rabbit monoclonal, Abcam AB32330), Calponin (1:250, rabbit monoclonal, Abcam AB46794), GFAP (1:1000, rabbit polyclonal, Dako Z0334), Map2ab (1:500, mouse monoclonal HM-2, Sigma), Nestin (1:1000, rabbit polyclonal, Chemicon AB5922), Nkx2.2 (1:40, mouse monoclonal 74.5A5, Developmental Biology Hybridoma Bank, University of Iowa), Nkx6.1 (1:100, mouse monoclonal F55A10, Developmental Biology Hybridoma Bank), Olig2 (1:500, rabbit polyclonal, IBL 18953), Snai2 (1:50, rabbit monoclonal, Cell Signalling C19G7), Twist1 (1:2, mouse monoclonal, Abcam AB50887). Cy3 or Cy5 conjugated-secondary antibodies (1:500, Invitrogen) were used.

### QPCR analysis

RNA extractions from spinal cord cells (passage 4) and human fetal brain neural stem cells (cultured in spinal cord cell medium for three passages) were performed using kits from Qiagen http://www.qiagen.com. Quantitative real-time RT-PCR was performed as described previously [38]. PCR was performed using the SYBR Green PCR Core Reagents Kit (Perkin-Elmer Applied Biosystems). The thermal cycling conditions comprised an initial denaturation step at 95°C for 10 min and 50 cycles at 95°C for 15 s and 65°C for 1 min. Experiments were performed with duplicates for each data point. Primer sequences are provided as additional file 1, Table S1. We quantified transcripts of the TBP gene (TATA binding protein) (Figure 1) or GAPDH (Figure 6) as the endogenous reference RNA control. Results, expressed as n-fold differences in target gene expression relative to the reference gene (termed Ntarget), were determined with the following formula: Ntarget = 2^ΔCt ^sample, where the ΔCt value of the sample was determined by subtracting the average Ct value of the target gene from the average Ct value of the reference gene.

### Western blot analysis

Protein preparation and Western blot procedure were described in [39]. Precision Plus Protein WesternC Standards were used (Bio-Rad).

## Authors' contributions

DM, CB, MT, DN, HG carried out cell culture, cell characterization and differentiation. CR, VR, BR performed spinal cord immunochemistry and western blot analyses. IB, RL carried out the QPCR analysis. LB, FEP carried out the spinal cord removal. AP participated in the design and coordination of the study. FV implemented the spinal cord removal procedure in the Montpellier Hospital. JPH designed and supervised the experiments and wrote the article.

All authors read and approved the final manuscript.

## Supplementary Material

Additional file 1 **Table S1: Table of primers used in QPCR analysis (Figure 1C, D)**. Click here for file
