# Supplementary material for: Isolation of mineralizing Nestin+ Nkx6.1+ vascular muscular cells from the adult human spinal cord
Source: BMC Neurosci. 2011 Oct 10;12:99. doi: 10.1186/1471-2202-12-99 (PMC3205052; doi:10.1186/1471-2202-12-99)
Supplement: Additional file 1 — Table S1: Table of primers used in QPCR analysis (Figure 1C, D). [file 1471-2202-12-99-S1.DOC]

| **Gene** | **Primer sequences** | |
| --- | --- | --- |
|  | **Left** | **Right** |
| **ACTA2** | 5'GGA CGC ACA ACT GGC ATC GTG 3' | 5' GAT GGC ATG GGG CAA GGC A 3' |
| **ALDH1L1** | 5'- CCG GCA TGG CTC CAT CAT CT -3' | 5'- TCT CCG TGA ATG AGG GTC CAG TT -3' |
| **ASCL1** | 5'- CGT CCT GTC GCC CAC CAT CT -3' | 5'- GGG GCT GAG CGG GTC GTA A -3' |
| **CD44** | 5'- CTT TCA ATA GCA CCT TGC CCA C -3' | 5'- CCC TTC TAT GAA CCC ATA CCT GC -3' |
| **CNP** | 5'- AGG AGT ACG CTC AAC AAG ATG TGT TA -3' | 5'- ACA AAG AGG GCA GAG ATG GTC A -3' |
| **COL1A1** | 5' CCT CCG GCT CCT GCT CCT CTT 3' | 5' GGC AGT TCT TGG TCT CGT CAC A 3' |
| **DCX** | 5'- AGC CAA GAG CCC TGG TCC TAT -3' | 5'- TGG AGG TTC CGT TTG CTG AGT -3' |
| **FABP7** | 5'- TCA TCA GGA CTC TCA GCA CAT TCA A -3' | 5'- CCA TCC AGG CTA ACA ACA GAC TTA CA -3' |
| **FN1** | 5'- TGT TGT CAC CAC TCT GGA GAA TGT -3' | 5'- GAA GCC AGT GAT CGT CTC AGT CTT -3' |
| **FOXC2** | 5'- GGA GTC CCA GGT GAG TGG CAA -3' | 5'- GAG AGG CGG CGT GGA TCT GTA -3' |
| **GFAP** | 5' CAG AAG CTC CAG GAT GAA ACC AA 3' | 5' GTG GCT TCA TCT GCT TCC TGT CT 3' |
| **MBP** | 5'- CCA AGT ACC TGG CCA CAG CAA GT -3' | 5'- GGA GTC AAG GAT GCC CGT GTC T -3' |
| **NEFL** | 5'- GAG ATT GCA GCT TAC AGG AAA CTC TT -3' | 5'- AGC TCT GGG AGT AGC CAC TGG T -3' |
| **NES** | 5' GAG GTG GCC ACG TAC AGG ACC 3' | 5' CTG AAA GCT GAG GGA AGT CTT GGA 3' |
| **NKX2.2** | 5'- CCT TCT ACG ACA GCA GCG ACA A -3' | 5'- GCC AGA CCG TGC AGG GAG T -3' |
| **NKX6.1** | 5'- GAG AGG GCT CGT TTG GCC TAT T -3' | 5'- CGG TTC TGG AAC CAG ACC TTG A -3' |
| **OLIG1** | 5'- CAC AGC GGC CCG GAG ACT T -3' | 5'- CCT GTA GCC CAC CAG CTC GTA GA -3' |
| **OLIG2** | 5' CGC CAG AGC CCG ATG ACC TT 3' | 5' GAC ACG GTG CCC CCA GTG AA 3' |
| **PDGFRA** | 5' CAT TTA CAT CTA TGT GCC AGA CCC A 3' | 5' ATG GCA GAA TCA TCA TCC TCC AC 3' |
| **PDGFRB** | 5' CCC CAG TGC CGA GTT AGA AGA C 3' | 5' GCA CGT AGC CGC TCT CAA CC 3' |
| **PLP1** | 5'- GAA GCC CTC ACT GGC ACA GAA -3' | 5'- ATA CTG GAA GGC ATG GAT CAC ATT -3' |
| **PROM1** | 5'- TGG TCC AAC AGG GCT ATC AAT C -3' | 5'- TTC AAG ACC CTT TTG ATA CCT GCT A -3' |
| **SNAI1** | 5'- GCT GCA GGA CTC TAA TCC AGA GTT -3' | 5'- GAC AGA GTC CCA GAT GAG CAT TG -3' |
| **SNAI2** | 5'- GCG AAC TGG ACA CAC ATA CAG TGA -3' | 5'- GCA GCG GTA GTC CAC ACA GTG A -3' |
| **SOX10** | 5' GCA AGC TCT GGA GGC TGC TGA 3' | 5' GGC GGC CTT CCC GTT CTT C 3' |
| **SOX2** | 5'- GCC CCC AGC AGA CTT CAC AT -3' | 5'- AGG GGC AGT GTG CCG TTA AT -3' |
| **TGFB1** | 5' GTC ACC CGC GTG CTA ATG GT 3' | 5' TTC TCG GAG CTC TGA TGT GTT GA 3' |
| **TWIST1** | 5' CGC CCC GCT CTT CTC CTC T 3' | 5' TGG ACA CGT CCT GCA TCA TCT CT 3' |
| **UGT8** | 5'- AAA GGC ATG GGG ATA TTG CTA GAA -3' | 5'- CCC TCT GAC GGT AGC TGG GAT -3' |
| **VIM** | 5'- CCT TGA ACG CAA AGT GGA ATC -3' | 5'- GAC ATG CTG TTC CTG AAT CTG AG -3' |
| **ZEB1** | 5'- ACA GTG TTA CCA GGG AGG AGC AGT -3' | 5'- TTT CTT GCC CTT CCT TTC TGT CAT -3' |
| **ZEB2** | 5'- GCC TCT GTA GAT GGT CCA GAA GAA -3' | 5'- TGT TAA TTG CGG TCT GGA TCG T -3' |
